# Supplementary material for: Toripalimab plus capecitabine in the treatment of patients with residual nasopharyngeal carcinoma: a single-arm phase 2 trial
Source: Nat Commun. 2024 Jan 31;15:949. doi: 10.1038/s41467-024-45276-1 (PMC10831082; doi:10.1038/s41467-024-45276-1)
Supplement: Supplementary file 1 — Supplementary Information [file 41467_2024_45276_MOESM1_ESM.pdf]

## Supplementary Information

## Supplementary Methods

**Description of the guidelines for intensity-modulated radiotherapy**

All eligible patients in the present trial received intensity-modulated radiotherapy.

In general, all patients were immobilised in the supine position with a thermoplastic mask used to cover the head, neck, and shoulder. Both non-enhanced computed tomography (CT) (for dose calculation) and contrast-enhanced CT (for target delineation) images) were obtained from the vertex to 2 cm below the sternoclavicular joint, with 3-mm slices.

The International Commission on Radiation Units and Measurements (ICRU) reports 50 and 62 were used to define the target volumes. We defined the gross tumour volume (GTV; including GTVnx and GTVnd) as the gross tumour determined by the physical examination, endoscopic findings, and imaging (including magnetic resonance imaging [MRI] and positron emission tomography [PET]-CT, if available) before induction chemotherapy, concurrent chemoradiotherapy, or radiotherapy. GTVnx represented the sum of the enlarged retropharyngeal nodes and the primary tumour volume, while GTVnd represented the volume of the involved gross cervical lymph nodes. The GTVnx plus a 5–10-mm margin (2–3 mm posteriorly if adjacent to the spinal cord or brain stem) defined the high-risk clinical target volume (CTV1), which included the whole nasopharynx and the high-risk sites of microscopic extension. The CTV1 plus a 5–10-mm margin (2–3 mm posteriorly if adjacent to the spinal cord or brain stem) defined the low-risk clinical target volume (CTV2), which encompassed the low-risk sites of microscopic extension, such as the retropharyngeal nodal regions, parapharyngeal space, clivus, foramen lacerum, sphenoid sinus, pterygopalatine fossae, oval foramen, pterygoid fossae, posterior parts of the nasal cavity, the cervical level containing the involved lymph nodes, the elective neck area from level II to Vb, and the supraclavicular fossae. Level Ib was subjected to electively irradiation if: (1) there was involvement of level Ib lymph nodes; (2) level IIa lymph nodes with extracapsular extension or a diameter ≥ 2 cm was noted or there was bilateral involvement; (3) the ipsilateral neck had extensive nodal disease; and (4) the soft or hard palate, oral cavity, or ipsilateral nasal cavity were grossly involved. A three-dimensional margin of 3–5 mm was added to the delineated target volume to compensate for internal organ motion and treatment set-up uncertainties, thus forming the planning target volume (PTV). The planning organ at risk volume (PRV) was formed by adding a 3-mm margin to the critical organs (eg, the spinal cord and brain stem).

The prescribed doses were 66–70 Gy, 64–70 Gy, 60–62 Gy, and 54–56 Gy, in 30–33 fractions, for the PTVs derived from GTVnx, GTVnd, CTV1, and CTV2, respectively. The radiation dose could be adjusted moderately according to the tumour volume. The normal tissue dose constraints are listed in the following table. A team of dosimetrists generated all the plans by employing a whole-field (including neck radiation) simultaneous integrated boost technique. In general, when critical normal tissues (eg, the brain stem and spinal cord) were adjacent to the high-dose target volumes, the target volume coverage could be compromised to keep these critical normal tissues within the dose constraints. When other normal tissues of lower priority were adjacent to the high-dose target volumes, the dose to these tissues was kept as low as possible without compromising the target coverage. The trade-off between covering the target volume and protecting the normal tissues in each case was discussed and decided upon by the research team. If the patients received induction chemotherapy, chemoradiotherapy or radiotherapy was recommended to commence within the first 21–28 days of the last induction chemotherapy cycle.

## Supplementary Methods

**Radiological diagnostic criteria of residual nasopharyngeal carcinoma[1-4]**

Diagnostic criteria of tumour residue were:

(1) Tumour residue in nasopharynx, other soft tissues or intracranial spaces, represented as hypo-intense signal on T1-weighted imaging, hyper-intense signal on T2-weighted imaging and enhancement following administration of Gd-DTPA;

(2) Skull base lesions were considered tumour residue if the bone of the skull base was destructed with soft tissues and the degree, and/or scope of bone strengthening had not decreased or increased compared to pre-treatment images;

(3) Regional lymph nodes were diagnosed as residual if they had a short-axis diameter >10 mm for cervical lymph nodes and >5 mm for retropharyngeal nodes, or if central necrosis was present.

## Supplementary Tables

**Supplementary Table 1. Administration of upfront induction chemotherapy ***

| | Patients (n=22) | |
|---|---|---|
| **Induction chemotherapy regimens** | | |
| Docetaxel, cisplatin, and fluorouracil | 10 | (45.5%) |
| Docetaxel and cisplatin | 5 | (22.7%) |
| Cisplatin and fluorouracil | 6 | (27.3%) |
| Lobaplatin and fluorouracil | 1 | (4.5%) |
| **Number of cycles of induction chemotherapy** | | |
| 2 cycles | 17 | (77.3%) |
| 3 cycles | 5 | (22.7%) |

Data are n (%). Percentages (%) might not total 100% because of rounding.

* 22 of 23 patients (95.7%) received induction chemotherapy.

**Supplementary Table 2. Regimens of induction chemotherapy**

| | | | | |
|---|---|---|---|---|
| **Regimen 1:** | | | | |
| Docetaxel | 60 mg/$m^2$ | intravenously | day 1 | Q3W |
| Cisplatin | 60 mg/$m^2$ | intravenously | day 1 | Q3W |
| Fluorouracil | 600 mg/$m^2$ per day | continuous 120-h intravenous infusion | days 1-5 | Q3W |
| **Regimen 2:** | | | | |
| Docetaxel | 75 mg/$m^2$ | intravenously | day 1 | Q3W |
| Cisplatin | 75 mg/$m^2$ | intravenously | day 1 | Q3W |
| **Regimen 3:** | | | | |
| Cisplatin | 100 mg/$m^2$ | intravenously | day 1 | Q3W |
| Fluorouracil | 800 mg/$m^2$ per day | continuous 120-h intravenous infusion | days 1-5 | Q3W |
| **Regimen 4:** | | | | |
| Lobaplatin | 30 mg/$m^2$ | intravenously | day 1 | Q3W |
| Fluorouracil | 800 mg/$m^2$ per day | continuous 120-h intravenous infusion | days 1-5 | Q3W |

The starting dose of each drug could be modified moderately according to patient tolerance, but should be at least 90% of the predefined dose. Dose reductions because of toxicities were permitted. However, those with a reduction by more than one level (to 75% of the predefined dose) would be excluded from the present study.

**Supplementary Table 3. Administration of concurrent chemoradiotherapy**

| | Patients (n=23) | |
|---|---|---|
| **Radiotherapy technique** | | |
| Intensity-modulated radiotherapy (IMRT) | 23 | (100%) |
| **Concurrent chemotherapy** | | |
| Regimens | | |
| Cisplatin | 22/23 | (95.7%) |
| Lobaplatin | 1/23 | (4.3%) |
| Administration | | |
| Once every 3 weeks | 23/23 | (100%) |
| Number of cycles | | |
| 2 cycles | 23/23 | (100%) |

Data are n (%). Percentages (%) might not total 100% because of rounding.

**Supplementary Table 4. Regimens of concurrent chemotherapy**

| | | | | |
|---|---|---|---|---|
| **Regimen 1:** | | | | |
| Cisplatin | 100 mg/m$^2$ | intravenously | day 1 | Q3W |
| **Regimen 2:** | | | | |
| Lobaplatin | 30 mg/m$^2$ | intravenously | day 1 | Q3W |

Dose reductions because of toxicities were permitted. However, those with a reduction by more than one level (to 75% of the predefined dose) would be excluded from the present study.

**Supplementary Table 5. Normal tissue dose constraints used for plan optimization**

| Structure | Dose constraints |
|---|---|
| Spinal cord | Dmax * ≤ 45 Gy |
| Spinal cord_PRV | D1 †≤ 50 Gy |
| Brain stem | Dmax ≤ 54 Gy |
| Brain stem_PRV | D1 ≤ 60 Gy |
| Optic nerves | Dmax ≤ 54 Gy |
| Optic nerves_PRV | D1 ≤ 60 Gy |
| Optic chiasm | Dmax ≤ 54 Gy |
| Optic chiasm_PRV | D1 ≤ 60 Gy |
| Temporal lobe | Dmax ≤ 60 Gy |
| Temporal lobe_PRV | D1 ≤ 65 Gy |
| Lens | Dmean ‡ < 8 Gy |
| Pituitary | Dmax < 60 Gy |
| Eyes | Dmean < 35 Gy |
| Mandible | Dmax < 70 Gy |
| Temporomandibular Joint | Dmax < 70 Gy |
| Parotid | Dmean < 26 Gy |
| Parotid | V30 § < 50% |
| Cochlea | Dmean < 50 Gy |
| Larynx | Dmean < 45 Gy |

PRV = planning organ at risk volume.

* Maximum point dose to the target volume.

† Dose received by 1% of the target volume.

‡ Mean dose to the target volume.

§ At least 50% of the gland will receive <30 Gy (should be achieved in at least one gland).

**Supplementary Table 6. Response to treatment according to previous exposure to fluorouracil**

| | Efficacy population (n=23) | | | | |
|---|---|---|---|---|---|
| | **Previous exposure to fluorouracil (n=17)** | | **Non- previous exposure to fluorouracil (n=6)** | | **p value** |
| Complete response | 8 | (47.1%) | 4 | (66.7%) | |
| Partial response | 8 | (47.1%) | 2 | (33.3%) | |
| Stable disease | 1 | (5.9%) | 0 | - | |
| Objective response rate | 16 | (94.1%; 71.3-99.9) | 5 | (100%; 100-100) | 1.000 * |

Data are n (%) or n (%; 95% CI). Percentages (%) might not total 100% because of rounding. 95% CI=95% confidence interval. *p values were calculated with Fisher's Exact $\chi^2$ test.

**Supplementary Table 7. Immune-related adverse events**

| | Any grade | | Grade 1 | | Grade 2 | |
|---|---|---|---|---|---|---|
| **Any adverse event** | 15 | (65.2%) | 10 | (43.5%) | 5 | (21.7%) |
| **Haematological adverse event** | | | | | | |
| Leukopenia | 3 | (13.0%) | 2 | (8.7%) | 1 | (4.3%) |
| Lymphopenia | 3 | (13.0%) | 1 | (4.3%) | 2 | (8.7%) |
| Anemia | 3 | (13.0%) | 2 | (8.7%) | 1 | (4.3%) |
| Neutropenia | 2 | (8.7%) | 1 | (4.3%) | 1 | (4.3%) |
| **Non-haematological adverse event** | | | | | | |
| Hypothyroidism | 6 | (26.1%) | 4 | (17.4%) | 2 | (8.7%) |
| Hyperthyroidism | 3 | (13.0%) | 3 | (13.0%) | 0 | |
| Fatigue | 3 | (13.0%) | 2 | (8.7%) | 1 | (4.3%) |
| Rash | 2 | (8.7%) | 1 | (4.3%) | 1 | (4.3%) |
| Pruritus | 2 | (8.7%) | 1 | (4.3%) | 1 | (4.3%) |
| Fever | 1 | (4.3%) | 1 | (4.3%) | 0 | |
| Proteinuria | 1 | (4.3%) | 1 | (4.3%) | 0 | |
| Hyperglycemia | 1 | (4.3%) | 1 | (4.3%) | 0 | |
| Thyroid stimulating hormone increased | 1 | (4.3%) | 1 | (4.3%) | 0 | |
| Myalgia | 1 | (4.3%) | 1 | (4.3%) | 0 | |

Data are n (%). Some patients had more than one adverse event. No grade 3-5 adverse events were reported.

**Supplementary Table 8. Compliance to treatment**

| | All patients enrolled (n=23) | |
|---|---|---|
| **Completed 6 cycles of scheduled treatment** | 20 | (87.0%) |
| **Discontinued scheduled treatment** | 3 | (13.0%) |
| **Toripalimab** | | |
| Number of patients received full dosage | 20 | (87.0%) |
| Relative dose intensity (%, median [IQR]) | 100% | (100-100) |
| **Capecitabine** | | |
| Number of patients received full dosage | 12 | (52.2%) |
| Relative dose intensity (%, median [IQR]) | 100% | (89.6-100) |

Data are n (%), or median (IQR). For each drug, the relative dose intensity was calculated as: total dose actually received/(predefined dose × cycles). IQR=interquartile range.

## Supplementary References


1 Lv, J. W. et al. Magnetic Resonance Imaging-Detected Tumor Residue after Intensity-Modulated Radiation Therapy and its Association with Post-Radiation Plasma Epstein-Barr Virus Deoxyribonucleic Acid in Nasopharyngeal Carcinoma. *J Cancer*. **8**, 861-869 (2017).

2 He, Y. et al. A retrospective study of the prognostic value of MRI-derived residual tumors at the end of intensity-modulated radiotherapy in 358 patients with locally-advanced nasopharyngeal carcinoma. *Radiat Oncol*. **10**, 89 (2015).

3 Lin, G. W., Wang, L. X., Ji, M. & Qian, H. Z. The use of MR imaging to detect residual versus recurrent nasopharyngeal carcinoma following treatment with radiation therapy. *Eur J Radiol*. **82**, 2240-2246 (2013).

4 Ng, S. H. et al. Comprehensive imaging of residual/ recurrent nasopharyngeal carcinoma using whole-body MRI at 3 T compared with FDG-PET-CT. *Eur Radiol*. **20**, 2229-2240 (2010).

**Supplementary Note**

# Study Protocol

# Toripalimab in combination with capecitabine as the salvage treatment for the patients with residual nasopharyngeal carcinoma: a prospective, single-arm, open-label, phase 2 trial

**Principal investigator:** Prof. Xiang Guo

Department of Nasopharyngeal carcinoma, Sun Yat-sen University Cancer Centre

**Version:** 2.2

Date: May 6, 2020

## 1. Background

Nasopharyngeal carcinoma is one of the most common cancer of the head and neck in southern China. Radiotherapy has become the main therapy for nasopharyngeal carcinoma due to the hidden anatomical sites, the large number of surrounding important organs, the poor differentiation of the tumour cells, and the high sensitivity of the tumour cells to radiotherapy. With updated radiotherapy technology and equipment, especially the promotion of intensity-modulated radiotherapy (IMRT) in clinical practice, the 5-year overall survival rate of early-stage nasopharyngeal carcinoma has reached >90%; however, the 5-year overall survival rate of locoregionally advanced disease has been hovering at 60%-80%. Many modalities have been applied to improve the local control rate of patients with locoregionally advanced nasopharyngeal carcinoma, including concurrent chemoradiotherapy and IMRT combined with radiotherapy sensitizers. [1-5] However, 6.7%-13% of patients with nasopharyngeal carcinoma have residual disease after primary radical treatment. [5-9]

Residual nasopharyngeal carcinoma after primary radical treatment indicates poor prognosis[10]. Using MRI of nasopharynx and neck, He et al re-examined 358 patients with locoregionally advanced nasopharyngeal carcinoma after the completion of IMRT. [11] Compared with the patients without MRI-indicated residual tumours, the patients with MRI-indicated residual tumours had significantly lower 3-year overall survival rate, 3-year local relapse-free survival rate, and 3-year disease-free survival rate. [11] Kwong et al. also found a progressive decline in 5-year local control rate (82% *vs* 77% *vs* 40%), 5-year disease-free survival rate (72% *vs* 69% *vs* 47%), and 5-year overall survival rate (79% *vs* 65% *vs* 54%) in patients who showed early tumour regression, delayed tumour regression, and residual tumours; with the latter population of patients having significantly lower 5-year local control rate, 5-year disease-free survival rate, and 5-year overall survival rate compared to former two populations ($p<0.001$). [12] Therefore, even though continued tumour regression is possible, patients with residual tumours have a worse prognosis than those with early and delayed tumour regression. The subsequent therapy will help to improve their prognoses.

The treatments for residual nasopharyngeal carcinoma include radiotherapy, surgery, chemotherapy, and photodynamic therapy. However, salvage treatments do not show satisfactory efficacy for residual diseases. There is no consensus on the optimal treatment, with higher incidences of various treatment-related complications and fatal effects. [13,14]

The side effects of re-irradiation are extremely serious in patients with residual nasopharyngeal carcinoma. After single-fraction stereotactic radiotherapy, the incidence of cerebral necrosis was 9%-25% and that of nasopharyngeal haemorrhage was 6%-8%, and death may also occur. [15,16] Chua et al. and Wu et al. showed no significant difference in the incidence of adverse effects after fractionated stereotactic radiotherapy versus single-fraction stereotactic radiotherapy. [15,16] The results of the aforementioned studies also suggested a higher incidence of nasopharyngeal haemorrhage in cases with residual diseases invading the cavernous sinus or carotid sheath, or accompanied by nasopharyngeal necrosis or internal carotid aneurysm. [15,16] Therefore, re-irradiation should be avoided when a residual disease is present in the conditions above. [17,18] Yau et al. used conventional two-dimensional radiotherapy to treat residual disease and observed a 17% incidence of radiation encephalopathy after treatment, compared to 6% in patients only treated with primary radiotherapy ($p<0.05$). [6] Zheng et al. used three-dimensional radiotherapy to treat residual disease and observed an incidence of late grade 3-4 toxicity as high as 16.7%.[7] IMRT can significantly increase the dose to the tumour while reducing the dose to normal tissues to mitigate radiation sequelae. However, because the nasopharyngeal mucosa is irradiated with a high total dose in primary treatment, serious complications such as nasopharyngeal mucosal necrosis and

haemorrhage may occur during or after re-irradiation, which also limits the use of IMRT in the treatment of residual nasopharyngeal carcinoma.

The surgery of residual nasopharyngeal carcinoma allows direct resection of residual tumours, thereby avoiding the toxicities of radiation. However, the complex structure of the nasopharynx makes surgery unsuitable when: (1) the residual tumours is located in the skull base bone, cavernous sinus, infratemporal fossa, or orbit; (2) the distance between the residual tumours and the internal carotid artery is <1 cm and, thus, an adequate surgical margin cannot be ensured; (3) the residual tumours invade the intracranial or internal carotid artery.[14,17,18]

The primary treatment for residual lymph nodes is cervical dissection; however, radical resection cannot be accomplished in patients with extranodal extension into the carotid sheath, residual lymph nodes located too high near the base of the skull, or residual retropharyngeal lymph nodes. [19,20] Chan et al. performed extended radical neck dissection in 158 nasopharyngeal carcinoma patients with residual or recurrent neck lymph nodes after radical radiotherapy (11 patients with residual neck lymph nodes and 147 patients with recurrent neck lymph nodes). 25.9% of patients with macroscopic extranodal extension were additionally treated with simultaneous skin flap grafting and postoperative brachytherapy. The investigators noted that the extranodal extension before surgery should not be too large in scope as the surgical wound will be large. The study compared the outcomes of patients with extranodal extension versus those without. The 5-year nodal control rates were 62% and 65% (p=0.18) and the 5-year disease-free survival rates were 38% and 44% (p=0.08), respectively. Furthermore, a high risk of postoperative complications (eg, carotid artery stenosis, vagus, and hypoglossal nerve palsy) were reported. [21]

Re-irradiation or surgery are recommended if the residual nasopharyngeal carcinoma is small and limited. Chemotherapy may play an important role in the treatment of residual disease, which are so extensive that cannot be effectively treated with re-irradiation or surgery. Of note, patients with nasopharyngeal carcinoma generally lose 5%-10% of their body weight and are in poor nutritional status after radical treatments due to treatment-related toxic effects. Therefore, these patients received intravenous chemotherapy may result in higher toxicity and worse efficacy. A study from Hong Kong, China, showed that only 19 of 319 patients (6%) with residual disease underwent a second cycle of intravenous chemotherapy, one of whom died owing to toxicity. [13] Compared to intravenous chemotherapy, oral chemotherapy offers benefits to patients due to its non-inferior effectiveness, less toxicities, and higher treatment compliance.

Capecitabine is a synthetic, orally administered precursor of 5-fluorouracil (5-FU). Compared with 5-FU, capecitabine has a stable and higher blood level. In addition, the convenience of oral administration also makes capecitabine a good alternative drug. The drug passes through the intestinal mucosa as a prototype and is sequentially metabolized by three enzymes, eventually converting to 5-FU under the action of thymidine phosphorylase in tumour cells, thereby reducing the incidence of adverse effects and improving therapeutic targeting. Capecitabine has shown significant efficacy in the first-line treatment of metastatic colorectal cancer. [22-24] Capecitabine also is recommended as a salvage drug for metastatic breast cancer that is resistant to other chemotherapeutic drugs. [25-27]. Chua et al. investigated the efficacy of capecitabine monotherapy in 49 patients with recurrent or metastatic nasopharyngeal carcinoma (55% patients with local recurrence), and found that the regimen achieved a complete response rate of 6% and a partial response rate of 31% (with an overall response rate of 37%), with a median progression-free survival of five months and a median overall survival of 14 months, respectively; the 1-year and 2-year overall survival rate were 54% and 26%, respectively. [28] In addition, Chua et al. showed that capecitabine monotherapy was well tolerated and had good compliance, with two patients had grade 3 anaemia and

one patient had grade 3 thrombocytopenia. The most common non-haematological toxicity manifested as hand-foot syndrome. [28]

In two clinical trials, the sequential administration of low-dose capecitabine (650 mg/m$^2$ or 800 mg/m$^2$, twice per day) achieved a comparable efficacy to the administration of high-dose capecitabine (1000 mg/m$^2$ or 1250 mg/m $^2$, days 1-14, every 3 weeks) in the treatment of metastatic breast cancer, [29,30] which provides a reasonable basis for a dose reduction of capecitabine. In the study by Chua et al., 37 patients (75.5%) were treated with capecitabine at a standard dose (1250 mg/m$^2$, twice per day), while starting dose of capecitabine was reduced to 1000 mg/m$^2$ as a high proportion of severe hand-foot syndrome. [28] In clinical practice, patients with nasopharyngeal carcinoma experience decreased fitness, severe oral mucositis, and malnutrition after initial radical treatment. Considering efficacy, safety, and compliance, the present clinical trial proposes a regimen of administering capecitabine at a dose of 1000 mg/m$^2$, twice daily from days 1-14 to patients with residual nasopharyngeal carcinoma after standard-of-care treatment.

While capecitabine has a relatively high overall response rate in nasopharyngeal carcinoma patients, the progression-free survival could be further improved. [28,31] Given the massive lymphocyte infiltration in nasopharyngeal carcinoma, immunotherapy may be an effective salvage therapy for this disease. Studies have shown a high expression (37.4%-48.7%) of programmed cell death-ligand 1 (PD-L1) in patients with nasopharyngeal carcinoma. Programmed cell death-l (PD-1) is an important immunosuppressive molecule mainly expressed on the surface of activated T cells; its ligand, PD-L1, is overexpressed on tumour cells and binds to PD-1 on the surface of T cells to inhibit the immunosurveillance function of T cells, thereby accelerating cancer cell proliferation and metastasis. [32,33] The prognoses of nasopharyngeal carcinoma patients with high PD-L1 expression is poor, [34-37] suggesting the potential efficacy of immunotherapy in the treatment for nasopharyngeal carcinoma.

Since 2014, an increasing number of anti-PD-1 antibodies and anti-PD-L1 antibodies have been developed, which have shown encouraging efficacy in a variety of solid tumour. [38-41] Toripalimab is first recombinant humanized anti-PD-1 monoclonal antibody in China. It is injectable and generated on a human IgG4/Kappa isotype backbone and has a stable structure with unique complementarity determining region (CDR) sequences and FG loops, showing low immunogenicity and no antibody-dependent cell-mediated or complement-dependent cytotoxic effects. Compared with nivolumab and pembrolizumab, toripalimab has a higher affinity for PD-1 and slower dissociation after binding to the PD-1, with an ability to promote T-lymphocyte proliferation and increase the proportion of memory effector T-lymphocytes while inducing endocytosis of the PD-1, decreasing PD-1 expression on the cell membrane surface, and enhancing the lymphocyte-killing effect. In a phase 1 trial, toripalimab was administered in three groups using a 3+3 dose escalation scheme with starting doses of 1 mg/kg, 3 mg/kg, and 10 mg/kg, respectively. The trial results showed: (1) at the highest exposure dose (10 mg/kg), no dose-limiting toxicity was observed and the maximum tolerated dose was not reached; (2) toripalimab had a relatively high anti-tumour activity for solid tumours (melanoma, transitional cell carcinoma, and renal cancer), leading to an objective response rate of 22% and a disease control rate of 53% with mostly grade 1-2 adverse effects (most commonly hypothyroidism). [42] Several phase 1/2 trials have shown that toripalimab has good therapeutic efficacy in a variety of tumours, including melanoma, urothelial carcinoma, head and neck cancer, non-small cell lung cancer, lymphoma, gastric adenocarcinoma, oesophageal squamous carcinoma, alveolar soft-part sarcoma, and other malignancies. [43-46]

In 2018, Xu et al. reported the results of a phase 2 trial for metastatic nasopharyngeal carcinoma at the European Society of Medical Oncology, [47] which enrolled a total of 139 patients with metastatic or refractory nasopharyngeal carcinoma after receiving standard chemotherapy. The enrolled patients

received toripalimab (3 mg/kg, every 3 weeks) until tumour progression or intolerable toxicity. The therapeutic efficacy was assessed every 8 weeks, and PD-L1 expression and plasma EBV-DNA levels were also monitored. By January 2018, the objective response rate was 30.8% and the disease control rate was 61.5% in 52 evaluable patients. The study also observed a 47-fold decrease in the replication rate of EBV-DNA in patients with disease response. Among the 139 patients, 84% experienced treatment-related adverse events, and 14.5% experienced grade 3 or higher adverse events, with most common adverse events being grade 1-2 (including fever [18.2%], hypothyroidism [18.2%], and proteinuria [10.9%]). The results showed that toripalimab leaded to promising efficacy, with a manageable safety profile in patients with metastatic nasopharyngeal carcinoma after multiple lines of chemotherapy. Furthermore, the copy number of plasma EBV-DNA might serve as a prognostic factor in nasopharyngeal carcinoma patients receiving immunotherapy.

Clinically, although capecitabine has been used for the treatment in residual nasopharyngeal carcinoma, the results have not been yet reported. On the other hand, while the efficacy and safety of toripalimab in recurrent or metastatic nasopharyngeal carcinoma have been confirmed by phase 2 trials, no published studies are available to support its efficacy and safety in patients with residual nasopharyngeal carcinoma after primary treatments. The anti-tumour mechanisms of toripalimab and capecitabine are different, and there are no significantly overlapping adverse events. Therefore, in this prospective, single-arm, open-label, phase 2 trial, we aim to assess the efficacy and safety of toripalimab plus capecitabine for patients with residual nasopharyngeal carcinoma after definitive treatment.

## 2. Objectives

The objective of this trial is to assess the efficacy and safety of toripalimab plus capecitabine for patients with residual nasopharyngeal carcinoma after definitive treatment.

## 3. Design

This is a prospective, single-arm, open-label, phase 2 trial conducted in an area with a high prevalence of nasopharyngeal carcinoma.

### 3.1. Endpoints

(1) Primary endpoint: objective response rate;

(2) Secondary endpoints: complete response rate, disease control rate, duration of response, progression-free survival, safety profile, treatment compliance;

### 3.2. Criteria of efficacy and safety

The efficacy will be evaluated according to Response Evaluation Criteria in Solid Tumours (RECIST, version 1.1). The safety will be evaluated according to National Cancer Institute Common Terminology Criteria for Adverse Events (NCI CTC-AE, version 5.0).

## 4. Trial subjects

### 4.1. Inclusion criteria

(1) Patients aged 18-70 years;

(2) Patients with histopathologically or cytologically confirmed World Health Organization (WHO) type II or type III nasopharyngeal carcinoma prior to primary treatment;

(3) Patients initially treated with radiotherapy, chemoradiotherapy, or induction chemotherapy followed by chemoradiotherapy;

(4) Patients with pathological， cytological, or radiological (at least two classic radiological features

[with or without clinical symptoms] on MRI, or PET-CT [if necessary]) diagnoses of residual nasopharyngeal carcinoma (nasopharyngeal tumours and/or neck lymph nodes) at 12–16 weeks after the completion of definitive treatment;

(5) Patients who able to undergo enhanced MRI;

(6) Patients not suitable for local treatment, where local treatment refers to methods related to anti-tumour therapy, including surgery and radiotherapy;

(7) Patients with an expected survival of ≥12 weeks;

(8) Patients with a Karnofsky Performance Scale (KPS) score ≥70;

(9) Fertile female patients with a negative serum pregnancy test result at screening (within seven days prior to the first administration of the trial drug) and taking effective contraception before entry into the trial and throughout the trial until six months after the last administration of the trial drug;

(10) Patients without serious dysfunctions of the heart, lung, liver, kidney, and other vital organs;

(11) Patients with normal liver and kidney functions: aspartate aminotransferase (AST) and alanine aminotransferase (ALT) ≤1.5 times the upper limit of normal (ULN), total bilirubin ≤1.5 times the ULN; creatinine clearance rate ≥60 mL/min; adequate bone marrow function: leukocyte count $>4.0\times10^9$/L, neutrophil count $>2.0\times10^9$/L, haemoglobin level >90 g/L, platelet count $>100\times10^9$/L;

(12) Patients who sign the informed consent form.

**4.2. Exclusion criteria**

(1) Patients with residual disease could not be identified and measurable on MRI;

(2) Patients with residual nasopharyngeal carcinoma who develop distant metastases prior to trial;

(3) Patients with a history of allergic to toripalimab, any component of capecitabine, or other monoclonal antibodies;

(4) Patients with prior treatments against the programmed cell death-1 (PD-1) receptor or its ligand PD-L1 or the cytotoxic T-lymphocyte-associated protein 4 (CTLA4) receptor, or received other immunotherapy;

(5) Patients who received any anti-tumour therapy within 4 weeks prior to the trial, or underwent major surgery, or had a history of serious trauma (except for primary treatment for nasopharyngeal carcinoma);

(6) Patients with a history of autoimmune disease, except for the following two types of patients after evaluation: (1) patients with autoimmune-associated hypothyroidism who are receiving stable doses of thyroid hormone replacement therapy; (2) patients with controlled type I diabetes mellitus on a stable insulin regimen;

(7) Patients administered a systemic immunostimulatory drug (including but not limited to interferon or interleukin [IL]-2) within 4 weeks prior to the trial or within 5 half-lives of the drug, whichever is shorter;

(8) Patients administered systemic corticosteroids (>10 mg/d prednisone or equivalent) or other systemic immunosuppressants within 2 weeks prior to the trial, except for patients using topical or inhaled corticosteroids;

(9) Patients with a history of bone marrow or organ transplantation;

(10) Patients with a history of idiopathic pulmonary fibrosis, drug-induced pneumonia, organizing pneumonia, or idiopathic pneumonia, or with other active pneumonia;

(11) Patients vaccinated within 4 weeks prior to the trial;

(12) Patients with active infectious disease, including tuberculosis, hepatitis B (hepatitis B surface

antigen [HBsAg]-positive), hepatitis C or AIDS (HIV antibody-positive);

(13) Patients unable to comply with treatment due to mental or other underlying illnesses;

(14) Female patients who are pregnant or lactation;

(15) Patients with major cardiovascular disease: patients with class II or higher New York Heart Association (NYHA) functional classification, with myocardial infarction within 1 year, with unstable angina, with supraventricular tachycardia, or with ventricular arrhythmia requiring clinical intervention;

(16) Patients unable to comply with regular follow-up visits for psychological, social, family, or geographical reasons;

(17) Patients with severe uncontrollable infections or medical conditions;

(18) Patients who suffer from major organ dysfunction such as decompensated cardiac, pulmonary, renal, or hepatic dysfunction and thereby are unable to tolerate treatment;

(19) Patients with the following laboratory test results: total bilirubin >1.5 times the ULN; AST and/or ALT >1.5 times the ULN with alkaline phosphatase >2.5 times the ULN;

(20) Patients with factors affecting drug administration, distribution, metabolism, and excretion, such as mental disorders, central nervous system disorders, chronic diarrhoea, ascites, and pleural fluid;

(21) Patients with other prior or concurrent malignancies (except for cured malignancies with disease-free survival of >5 years [eg, basal cell carcinoma of the skin, carcinoma in situ of the cervix]);

(22) Patients unwilling to sign the informed consent form.

### 4.3. Withdrawal criteria

(1) Physicians believe that the termination of study treatment is beneficial to patients according to their conditions;

(2) Patients who opt to withdraw from the trial;

(3) Patients unable to tolerate toxicity;

(4) Patients who fail to follow the treatment plan;

(5) Patients who participate in other chemotherapy, surgery, or experimental drug therapy during the trial.

## 5. Schema and implementation

### 5.1. Baseline assessments

(1) Medical history, coexisting illness, and concomitant medications;

(2) Vital signs, physical examination, weight, height, and Karnofsky Performance Scale (KPS) score;

(3) Haematological test, urine test, stool test, haemostasis and coagulation tests, and pregnancy test (for female).

(4) Biochemical test (liver function, kidney function, blood lipids, blood glucose, electrolytes), creatinine clearance, thyroid function (thyroid-stimulating hormone [TSH], free T3, free T4), serology for infectious diseases (HIV, hepatitis B virus [HBV], hepatitis C virus [HCV]), myocardial injury markers (troponin, B-type natriuretic peptide [BNP], cardiac enzymes);

(5) Plasma EBV-DNA;

(6) Electrocardiogram, echocardiography;

(7) Nasal endoscopy or rhino-sinusal endoscopy;

(8) Biopsy or fine needle aspiration of residual nasopharyngeal carcinoma;

(9) Histopathology;

(10) Enhanced MRI of the nasopharynx and neck;

(11) CT scan of the chest;

(12) Ultrasonography or CT scan of the abdomen;

(13) Bone scan and positron emission tomography-computed tomography (PET-CT) (if necessary);

**5.2. Staging of nasopharyngeal carcinoma**

American Joint Committee on Cancer staging system (AJCC, 8th edition)

**5.3. Treatment Plan**

Eligible patients will be treated with toripalimab plus capecitabine.

Toripalimab plus capecitabine will be administered once every 3 weeks until disease progression, intolerable drug side effects, or the completion of six cycles of treatment.

*(Shanghai Junshi Biosciences Co. offered toripalimab free of charge to enrolled patients in this trial*)

**5.3.1. Medication regimen**

The combination of toripalimab and capecitabine therapy:

| Toripalimab: | 240 mg | Once per day | Intravenously | Day 1 | Every 3 weeks |
|---|---|---|---|---|---|
| | (at least 60 minutes for the first dose; the time may be shortened to 30 minutes for the second dose) | | | | |
| Capecitabine: | 1000 $mg/m^2$ | Twice per day | Orally | Days 1-14 | Every 3 weeks |

**5.3.2. Concomitant medication and treatment**

(1) Permitted treatments: topical application of steroids such as ocular, nasal, and inhalation steroids; supportive treatments (eg, pain treatment, intravenous nutrition treatment);

(2) Forbidden treatments: the administration of other experimental drugs or any other anti-cancer therapy during the trial, including immunomodulators such as thymosin and lentinan; long-term systemic glucocorticoid administration is prohibited except to mitigate immune-related adverse events.

**5.3.3. Monitoring toxicity, dose modification and interruption, and treatment discontinuation**

(1) Criteria for toxicity

NCI CTC-AE (version 5.0).

(2) Dose modification

**<u>Capecitabine</u>**

According to the toxicity evaluation criteria, dose delay or modification will be assessed. Any patient with a dose reduction will continue to receive the reduced dose subsequently. In order to recover from toxicities, the treatment can be delayed until days 35 after day 1 of the cycle (ie, a 2-week delay in scheduled treatment). If a patient fails to resume the treatment on days 35 from the date of the last dose, the patient must withdraw from the trial unless specific permission is obtained from the investigator.

The dose modification of capecitabine is as follows:

No dose modification will be recommended in the grade 1 adverse event. In grade 2 or 3 adverse event, capecitabine should be interrupted. Once the adverse event recovery to grade 1, treatment will be resumed with the original or reduced dose, as shown in the table below. In grade 4 adverse event, treatment should be interrupted until recovery to grade 1, and then treatment will be resumed at 50% of the original dose. The missed dose of capecitabine due to toxic effects will not be administered, and the patient will continue the planned treatment.

Dose modification for hand-foot syndrome:

| Grade | Treatment | Dose modification (% original dose) |
|---|---|---|
| Grade 1 | Original dose | Original dose |
| Grade 2 | | |

| | | |
|---|---|---|
| 1st occurrence | Interruption until recovery to grade 0-1 | 100% |
| 2nd occurrence | Interruption until recovery to grade 0-1 | 75% |
| 3rd occurrence | Interruption until recovery to grade 0-1 | 50% |
| 4th occurrence | Permanent termination of treatment | - |
| Grade 3 | | |
| 1st occurrence | Interruption until recovery to grade 0-1 | 75% |
| 2nd occurrence | Interruption until recovery to grade 0-1 | 50% |
| 3rd occurrence | Permanent termination of treatment | - |

*Note: Patients experiencing discomfort due to hand-foot syndrome will be managed with topical emollients, weak topical steroids, or urea-containing creams. Treatment will be continued in the event of toxic effects which are not associated with study medications.*

Dose modification for other toxicities (including haematological and non- haematological toxicities):

| Grade | Treatment | Dose modification (% original dose) |
|---|---|---|
| Grade 1 | Original dose | Original dose |
| Grade 2 | | |
| 1st occurrence | Interruption until recovery to grade 0-1 | 100% |
| 2nd occurrence | Interruption until recovery to grade 0-1 | 75% |
| 3rd occurrence | Interruption until recovery to grade 0-1 | 50% |
| 4th occurrence | Permanent termination of treatment | - |
| Grade 3 | | |
| 1st occurrence | Interruption until recovery to grade 0-1 | 75% |
| 2nd occurrence | Interruption until recovery to grade 0-1 | 50% |
| 3rd occurrence | Permanent termination of treatment | - |
| Grade 4 | | |
| 1st occurrence | Permanent termination of treatment, or interruption until recovery to grade 0-1 if the physician believes it is in the patient's best interest to continue treatment | 50% |

**Toripalimab**

In previous trials of toripalimab, atypical responses (eg, temporary enlargement of the tumour or appearance of small new lesions within the first few months of treatment, and then tumour shrinkage) have been observed. If the patient has stable disease or reduced clinical symptoms, even if there is preliminary evidence of disease progression, toripalimab administration may be continued based on a judgment of overall clinical benefit until confirmed disease progression.

Depending on the safety and tolerability in the patients, administration may need to be interrupted or permanently discontinued. Dose modification will not be recommended. Interruption or permanent discontinuation of toripalimab are detailed in the following table.

| Immune-related adverse effects | Grade | Treatment |
|---|---|---|
| Pneumonia | Grade 2 | Interruption until recovery to grade 0-1 |

| | | |
|---|---|---|
| | Grade 3-4 or recurrent grade 2 | Permanent discontinuation of treatment |
| Diarrhoea or colitis | Grade 2-3 | Interruption until recovery to grade 0-1 |
| | Grade 4 | Permanent discontinuation of treatment |
| Hepatitis | Grade 2 (AST or ALT at 3-5 times the ULN or total bilirubin at 1·5-3 times the ULN) | Interruption until recovery to grade 0-1 |
| | Grade 3-4 (AST or AT >5 times the ULN or total bilirubin >3 times the ULN) | Permanent discontinuation of treatment |
| Nephritis | Grade 2-3 serum creatinine elevation | Interruption until recovery to grade 0-1 |
| | Grade 4 serum creatinine elevation | Permanent discontinuation of treatment |
| Endocrine disorders | Symptomatic grade 2-3 hypothyroidism;<br>Grade 2-3 hyperthyroidism;<br>Grade 2-3 hypophysitis;<br>Grade 2 adrenal dysfunction;<br>Grade 3 hyperglycaemia or type I diabetes | Interruption until recovery to grade 0-1 |
| | Grade 4 hypothyroidism;<br>Grade 4 hyperthyroidism;<br>Grade 4 hypophysitis;<br>Grade 3–4 adrenal dysfunction;<br>Grade 4 hyperglycaemia or type I diabetes | Permanent discontinuation of medication |
| Skin disorders | Grade 3 rash | Interruption until recovery to grade 0-1 |
| | Grade 4 rash;<br>Stevens-Johnson Syndrome (SJS) or toxic epidermal necrolysis (TEN) | Permanent discontinuation of medication |
| Thrombocytopenia | Grade 3 | Interruption until recovery to grade 0-1 |
| | Grade 4 | Permanent discontinuation of medication |
| Other | Grade 3-4 serum amylase elevation or lipase elevation;<br>Grade 2-3 pancreatitis;<br>Grade 2 myocarditis *;<br>Other immune-related grade 2-3 adverse events occurring for the first time | Interruption until recovery to grade 0-1 |
| | Grade 4 or recurrent pancreatitis of any grade;<br>Grade 3-4 myocarditis;<br>Grade 3-4 encephalitis; | Permanent discontinuation of medication |

| | | |
|---|---|---|
| | Other immune-related grade 4 adverse effects that occur for the first time | |
| Recurrent or persistent adverse events | Recurrent grade 3-4 adverse events (except endocrine disease);<br>Grade 2-3 adverse events that do not recovery to grade 0-1 (except endocrine disease) within 12 weeks of the last dose;<br>Failure to reduce corticosteroids to ≤10 mg/day (prednisone equivalent dose) within 12 weeks of last dose | Permanent discontinuation of medication |
| Infusion reaction | Grade 2 | Lowered drip rate or interruption; dose resumption can be considered when the symptoms have resolved |
| | Grade 3-4 | Permanent discontinuation of treatment, and symptomatic treatment of the infusion reaction |

* The safety of restarting treatment after myocarditis recovery to grade 0-1 is uncertain.

## 6. Assessment during and after the treatment (follow-up phase)

### 6.1. Assessment during the study treatment

| Time | Items |
|---|---|
| Each cycle (day 1, day 8) | Patient complaints (discomfort: toxicity assessment), weight, physical examination, KPS score;<br>Laboratory tests: haematological test |
| Each cycle (day 1) | Laboratory tests: biochemical test (liver function, kidney function, blood lipids, blood glucose, electrolytes), urine test, stool test, haemostasis and coagulation tests, thyroid function (TSH, free T3, free T4), myocardial injury markers (troponin, BNP, cardiac enzymes)<br>Nasal endoscopy or rhino-sinusal endoscopy<br>Blood samples |
| Completion of study treatment | Enhanced MRI of the nasopharynx and neck |

### 6.2. Assessment after the study treatment (follow-up phase)

A dedicated team will be responsible for follow-up. In order to accurately evaluate patient outcomes and toxicities, follow-up will be conducted through telephone calls and messages based on the outpatient and inpatient records of the physicians. The follow-up content will be managed according to the guidelines of nasopharyngeal carcinoma.

The details of the follow-up regimen are as follows:

| Time | |
|---|---|
| One month after completion of study treatment | Once follow-up |
| 1-3 years after completion of study treatment | Follow-up every 3 months |
| 4-5 years after completion of study treatment | Follow-up every 6 months |

| | |
|---|---|
| >5 years after completion of study treatment | Follow-up per year |

Follow-up content:

(1) Patient complaint;

(2) Weight, physical examination, and KPS score;

(3) Haematological test, biochemical test (liver function, kidney function, blood lipids, blood glucose, electrolytes), urine test, stool test, haemostasis and coagulation tests, thyroid function (TSH, free T3, free T4), myocardial injury markers (troponin, BNP, cardiac enzymes);

(4) Nasal endoscopy or rhino-sinusal endoscopy;

(5) Enhanced MRI of the nasopharynx and neck;

(6) CT or X-ray of the chest, ultrasonography or CT scan of the abdomen;

(7) Bone scan and PET-CT (if necessary).

### 6.3. Recording of related events during the follow-up phase

Progressive disease will be determined using histopathological/cytological diagnoses or imaging diagnosis. Tumour recurrence or metastasis will be confirmed based on the results of biopsy, fine needle aspiration or surgical specimen. For lesions that are not accessible, the clinical diagnosis will also be accepted by the investigators on the basis of the presence of at least two classic radiological features on CT, MRI, abdominal ultrasonography, or PET-CT (with or without clinical symptoms). Even in participants with long-term progression-free survival, MRI showing skull base bone destruction may persist. It is necessary to combine clinical features and laboratory/imaging examinations (including symptoms, signs, plasma EBV-DNA load, PET-CT, and dynamic changes of MR images) to determine whether progressive disease has occurred. Records should be kept of the dates of diagnosis of progressive disease; the sites should also be recorded.

All patients will be followed up until death and the cause of death should be recorded. Deaths due to unknown cause are counted as death due to nasopharyngeal carcinoma if disease is still present at last assessment.

## 7. Endpoint evaluation and definitions

### 7.1. Endpoints

#### 7.1.1. Primary endpoint

Objective response rate (completion of six cycles of scheduled treatment): the proportion of patients with confirmed complete response or partial response.

#### 7.1.2. Secondary endpoints

(1) Complete response rate: the proportion of patients who have complete response;

(2) Disease control rate: the proportion of patients who achieve an objective response or stable disease;

(3) Duration of response: time from first documented objective response to disease progression or death from any cause, whichever occur first;

(4) Progression-free survival: the time from treatment initiation to disease progression or death from any cause, whichever occur first;

(5) Safety profile;

(6) Treatment compliance

### 7.2. Criteria for evaluation

#### 7.2.1. Efficacy criteria

The efficacy will be assessed by an independent review team according to RECIST (version 1.1)

As nasopharyngeal tumour and their cervical lymph node metastases with extranodal extension are irregular, the measurement of diameter remains controversial. By first delineating the tumour and then measuring the diameter, the efficacy will be evaluated in this trial. Specifically, the steps are: (1) after primary radical treatment, using MRI of nasopharynx and neck, the persistent or residual nasopharyngeal carcinoma will be identified; (2) after pathological and/or radiological diagnosis of persistent or residual nasopharyngeal carcinoma, T1-enhanced MR images will be imported into Medical Imaging Interaction Toolkit (version 2017.07) and two senior physicians (>5 years of experience) will perform layer-by-layer delineation of the disease independently; (3) after the completion of delineation, a third senior physician will compare the outlines and then determine the outline of each patient (discussion and verification with the first two physicians if necessary); (4) the diameters of each delineated disease in three directions will be calculated using a diameter calculation program, and these calculated diameters will be used as the baseline diameters; (5) MRI of nasopharynx and neck will be performed in patients after completion of study treatment, followed by diameter measurement as described above. The efficacy will be evaluated using the results of diameter measurement.

#### 7.2.2. Safety criteria

NCI CTC-AE (version 5.0)

#### 7.2.3. See 5. and 6.

### 7.3. Evaluation timeline

| | |
|---|---|
| Objective response rate<br>Completer response rate<br>Disease control rate | The end of 3 cycles of scheduled treatment<br>Completion of s 6 cycle of scheduled treatment |
| Duration of response<br>Progression-free survival | The treatment and the follow-up phase; (see 6.) |
| Safety profile<br>Treatment compliance | The treatment phase; (see 5. and 6.) |

## 8. Safety

### 8.1. Adverse events

Adverse events refer to any adverse medical events that occur on the patients. They do not necessarily have a causal relationship with treatment. Investigators should keep a detailed record of any adverse events that occur in the patients. The record of adverse events shall include a description of the adverse events, the time of occurrence, severity, duration, measures taken, the results and outcomes. Investigators should assess the possible association between the adverse events and the study medications according to the five-level classification of "positive relevance, possible irrelevance, positive irrelevance, and inability to determine."

### 8.2. Criteria for evaluation

NCI CTC-AE (version 5.0)

### 8.3. Serious adverse events

(1) Serious adverse events include: death within 30 days after receiving the study drugs, or death caused by delayed toxicity of the study drugs 30 days later; grade 3 or 4 toxicities that are considered life-threatening or require hospitalization for 7 days or more; those that cause permanent disability or dysfunction; that lead to secondary tumors; cause reactions to drug overdoses; or other unpredictable adverse drug reactions;

(2) The following conditions do not need to be reported as serious adverse events: death caused by

cancer progression; hospitalization for chemotherapy-related toxicity, such as bone marrow suppression, fever, nausea, vomiting, etc.; secondary hospitalization because of the tumor, such as weight loss, fatigue, electrolyte disturbance, pain treatment, anxiety, and palliative treatment; and planned hospitalisation;

(3) Reporting system for serious adverse events: this trial will adopt a centralized safety collection with addresses for safety reporting. Once a serious adverse event is identified, it should be immediately reported to the principal investigator (Prof. Xiang Guo; Tel: 86-20-87343359; Fax: 86-20-87343359), the corresponding ethics committee, the study centre (Sun Yat-sen University Cancer Centre), and the national health authorities within 24 hours, and recorded on the case report form. With the participation of key investigators, appropriate measures should be taken quickly. Toxic reactions and deaths that occur 30 days after the end of the trial do not need to be reported if it can be clearly determined that they have nothing to do with the treatment.

### 8.4. Records

Patient complaints of discomfort and the results of relevant tests such as vital signs, laboratory tests, and imaging will be recorded. Adverse events that lead to treatment interruption or discontinuation will be recorded until they disappear. Pre-existing adverse events that have changed in grade will also be recorded. Any adverse event that occurs during the treatment will be clearly documented for each patient.

## 9. Data collection and monitoring

### 9.1. Case report form

A case report form will be used to record clinical data in this clinical trial. All relevant information of the patient in the trial should be recorded in a timely and true manner. As original material, the case report form should not be changed at will. The investigators should sign and date when it is necessary to correct the data. The case report forms will be triplicated and should be handed over to the statisticians, investigators, and sponsors for storage after trial.

*Note: All information for each patient will be recorded on the case report form, including the patient disease status before, during, and after the treatment. The information should also include toxicity, disease-related symptoms, efficacy, and dose modifications.*

### 9.2. Database creation

After receiving the case report forms, the data administrator will check the data and feedback possible questions. The investigators should verify the problem and respond as soon as possible. Then, the data administrator will establishe a database in time and double inputs the data. The database will be locked by the principal investigator, data administrator, and statistician, and must be backed up. To ensure data security, irrelevant personnel cannot access and modify the trial data. Any data changes need to be approved by the principal investigator, statistician, and data administrator.

### 9.3. Data regulation

All information about the enrolled patients after registration will be sent to Sun Yat-sen University Cancer Centre for management. The investigators have stewards taking charge of database management, and the data platform allows simultaneous input and double checking.

## 10. Statistical analysis

### 10.1. Calculation of sample size

Sample size calculation was performed by statisticians.

Combination of toripalimab and capecitabine therapy:

The sample size was calculated using Simon's Two-Stage design,[48,49] with a one-sided type I error rate (α) of 0.025 and a power of 0.8. The reported objective response rate of recurrent nasopharyngeal carcinoma patients receiving with capecitabine monotherapy was 47.8%.[31] We expect that the objective response rate for toripalimab and capecitabine combination treatment will be 80% in patients with persistent or residual nasopharyngeal carcinoma. Under these assumptions, the trial will be divided into two stages. Six patients will be treated in the first stage of this trial. The trial will be continued if more than three responses are observed in the first stage. Additional 15 patients will be enrolled in the second stage. If 15 responses (including the responses in the first stage) are observed, the study treatment will be considered a success. Considering a 10% lost-to-follow-up rate, a total of 23 patients will be required.

### 10.2. Population analysis

(1) Efficacy population：all patients who enrolled and received at least one dose of study medications.

(2) Safety population： all patients who enrolled and received at least one dose of study medications.

*Note: The patients who did not have at least one post-baseline efficacy assessment will be ruled out from efficacy population. The safety population will exclude the patients without any safety data.*

### 10.3. Analytical approach

The clinical and demographic characteristics of the patients will be summarised with descriptive statistics. Continuous variables will be described with their distribution range, mean, median, standard deviation (SD), and interquartile range (IQR) as appropriate. Categorical variables will be analysed by the number of occurrences and the incidence rate (%).

Efficacy analysis will be assessed based on the efficacy population. The objective response rate, complete response rate, and disease control rate will be calculated, and the accompanying 95% confidence intervals (95% CIs) will be calculated based on the Clopper-Pearson method. Median duration of response and median progression-free survival will be estimated using the Kaplan-Meier method, and the corresponding 95% CIs will be estimated using the Brookmeyer-Crowley method.

Safety analysis will be assessed based on the safety population. The numbers and incidences of each adverse event will be detailed in tabular form.

Study duration will be calculated from the first dose date to the last dose date. Relative dose intensity will be calculated as actual cumulative dose against planned total dose.

### 10.4. Statistical software

All analyses will be performed using SPSS (version 26.0), R (version 4.0.2), and SAS (version 9.4).

### 10.5. Monitoring

To monitor the trial and make decisions with respect to possible early stop and publication, we will appoint an Independent Data Monitoring Committee.

## 11. Ethical considerations

### 11.1. Ethical norms

This trial must comply with the Helsinki Declaration, the Drug Clinical Trial Management Code issued by the National Medical Products Administration of China, and related regulations. Before the commencement of this trial, the approval of the ethics committee of Sun Yat-sen University Cancer Centre must be obtained. During the trial, any changes made to this trial protocol will be reported to the Ethics Committee and placed on record.

### 11.2. Informed consent

Patients must provide informed consent to participate in this trial before receiving study treatment to protect the legitimate rights and interests of the patients. It is the responsibility of the investigators to

provide the subject, or his or her designated representative, with a complete and comprehensive description of the purpose of the trial, the effects of the drug, the possible side effects, and possible risks, and to inform the subject of their rights. Conversation is a very important part of the informed consent process. If the subject and his or her legal representative are illiterate, the informed consent process shall be attended by a witness, who shall sign the informed consent form after oral consent by the subject or his or her legitimate representative. A copy of the informed consent form and the contact information for the investigator and the ethics committee must be provided to the patient on request.

### 11.3. Emergency measures

The hospital must be equipped with the necessary medical rescue equipment, first aid drugs, and emergency measures.

## 12. Management of trial drugs

The management, distribution, and recovery of clinical drugs in this trial shall be the responsibility of designated investigators. The investigators must ensure that all trial drugs are used only for patients participating in this trial, that their doses and usage are in accordance with the trial scheme, and that the remaining drugs are returned to the manufacturer. Study drugs shall not be transferred to any non-trial participant.

## 13. Quality control

(1) Establishing various Standard Operating Procedures for this trial;
(2) Establishing standardized evaluation methods (including criteria for diagnosis, efficacy, and toxicities);
(3) Formulating a "statistical plan" by statisticians;
(4) Obtaining Good Clinical Practice (GCP) training certificates for key investigators;
(5) Training all investigators before the start of the trial;
(6) Appointing quality controllers and formulating quality control plans for regular inspection;
(7) Performing inspections and receiving the supervision of the central inspector;
(8) Setting up a coordinating committee and curative effect evaluation and follow-up group.

## References

1 Chang, J. T. et al. Locally recurrent nasopharyngeal carcinoma. *Radiother Oncol*. **54**, 135-142 (2000).
2 Xiao, J. P. & Xu, G. Z. Stereotactic radiotherapy--an approach to improve local control of nasopharyngeal carcinoma. *Chin J Cancer*. **29**, 123-125 (2010).
3 Yau, T. K. et al. Induction chemotherapy with cisplatin and gemcitabine followed by accelerated radiotherapy and concurrent cisplatin in patients with stage IV(A-B) nasopharyngeal carcinoma. *Head Neck*. **28**, 880-887 (2006).
4 Low, J. S., Chua, E. T., Gao, F. & Wee, J. T. Stereotactic radiosurgery plus intracavitary irradiation in the salvage of nasopharyngeal carcinoma. *Head Neck*. **28**, 321-329 (2006).
5 Leung, T. W. et al. Treatment results of 1070 patients with nasopharyngeal carcinoma: an analysis of survival and failure patterns. *Head Neck*. **27**, 555-565 (2005).
6 Yau, T. K. et al. Effectiveness of brachytherapy and fractionated stereotactic radiotherapy boost for persistent nasopharyngeal carcinoma. *Head Neck*. **26**, 1024-1030 (2004).
7 Zheng, X. K., Chen, L. H., Chen, Y. Q. & Deng, X. G. Three-dimensional conformal

radiotherapy versus intracavitary brachytherapy for salvage treatment of locally persistent nasopharyngeal carcinoma. *Int J Radiat Oncol Biol Phys*. **60**, 165-170 (2004).

8 Leung, T. W. et al. Salvage brachytherapy for patients with locally persistent nasopharyngeal carcinoma. *Int J Radiat Oncol Biol Phys*. **47**, 405-412 (2000).

9 Chua, D. T., Sham, J. S., Kwong, P. W., Hung, K. N. & Leung, L. H. Linear accelerator-based stereotactic radiosurgery for limited, locally persistent, and recurrent nasopharyngeal carcinoma: efficacy and complications. *Int J Radiat Oncol Biol Phys*. **56**, 177-183 (2003).

10 Mantyla, M., Kortekangas, A. E., Valavaara, R. A. & Nordman, E. M. Tumour regression during radiation treatment as a guide to prognosis. *Br J Radiol*. **52**, 972-977 (1979).

11 He, Y. et al. A retrospective study of the prognostic value of MRI-derived residual tumors at the end of intensity-modulated radiotherapy in 358 patients with locally-advanced nasopharyngeal carcinoma. *Radiat Oncol*. **10**, 89 (2015).

12 Kwong, D. L. et al. The time course of histologic remission after treatment of patients with nasopharyngeal carcinoma. *Cancer*. **85**, 1446-1453 (1999).

13 Yu, K. H. et al. Survival outcome of patients with nasopharyngeal carcinoma with first local failure: a study by the Hong Kong Nasopharyngeal Carcinoma Study Group. *Head & neck*. **27**, 397-405 (2005).

14 Wei, W. I., Chan, J. Y., Ng, R. W. & Ho, W. K. Surgical salvage of persistent or recurrent nasopharyngeal carcinoma with maxillary swing approach - Critical appraisal after 2 decades. *Head & neck*. **33**, 969-975 (2011).

15 Chua, D. T., Wu, S. X., Lee, V. & Tsang, J. Comparison of single versus fractionated dose of stereotactic radiotherapy for salvaging local failures of nasopharyngeal carcinoma: a matched-cohort analysis. *Head & neck oncology*. **1**, 13 (2009).

16 Xiao, J., Xu, G. & Miao, Y. Fractionated stereotactic radiosurgery for 50 patients with recurrent or residual nasopharyngeal carcinoma. *International journal of radiation oncology, biology, physics*. **51**, 164-170 (2001).

17 Chen, M. Y. et al. Endoscopic nasopharyngectomy for locally recurrent nasopharyngeal carcinoma. *The Laryngoscope*. **119**, 516-522 (2009).

18 Vlantis, A. C. et al. Nasopharyngectomy: does the approach to the nasopharynx influence survival? *Otolaryngology--head and neck surgery : official journal of American Academy of Otolaryngology-Head and Neck Surgery*. **139**, 40-46 (2008).

19 Ferlito, A. et al. Prognostic significance of microscopic and macroscopic extracapsular spread from metastatic tumor in the cervical lymph nodes. *Oral Oncol*. **38**, 747-751 (2002).

20 Chao, K. S., Wippold, F. J., Ozyigit, G., Tran, B. N. & Dempsey, J. F. Determination and delineation of nodal target volumes for head-and-neck cancer based on patterns of failure in patients receiving definitive and postoperative IMRT. *International journal of radiation oncology, biology, physics*. **53**, 1174-1184 (2002).

21 Chan, Y. W., Lee, V. H., Chow, V. L., To, V. S. & Wei, W. I. Extracapsular lymph node spread in recurrent nasopharyngeal carcinoma. *The Laryngoscope*. **121**, 2576-2580 (2011).

22 Van Cutsem, E. et al. Capecitabine, an oral fluoropyrimidine carbamate with substantial activity in advanced colorectal cancer: results of a randomized phase II study. *J Clin Oncol*. **18**, 1337-1345 (2000).

23 Van Cutsem, E. et al. Oral capecitabine compared with intravenous fluorouracil plus leucovorin in patients with metastatic colorectal cancer: results of a large phase III study. *J Clin Oncol*. **19**,

4097-4106 (2001).

24 Hoff, P. M. et al. Comparison of oral capecitabine versus intravenous fluorouracil plus leucovorin as first-line treatment in 605 patients with metastatic colorectal cancer: results of a randomized phase III study. *J Clin Oncol*. **19**, 2282-2292 (2001).

25 Blum, J. L. et al. Multicenter phase II study of capecitabine in paclitaxel-refractory metastatic breast cancer. *J Clin Oncol*. **17**, 485-493 (1999).

26 Blum, J. L. et al. Multicenter, Phase II study of capecitabine in taxane-pretreated metastatic breast carcinoma patients. *Cancer*. **92**, 1759-1768 (2001).

27 Talbot, D. C. et al. Randomised, phase II trial comparing oral capecitabine (Xeloda) with paclitaxel in patients with metastatic/advanced breast cancer pretreated with anthracyclines. *Br J Cancer*. **86**, 1367-1372 (2002).

28 Chua, D., Wei, W. I., Sham, J. S. & Au, G. K. Capecitabine monotherapy for recurrent and metastatic nasopharyngeal cancer. *Jpn J Clin Oncol*. **38**, 244-249 (2008).

29 Stockler, M. R. et al. Capecitabine versus classical cyclophosphamide, methotrexate, and fluorouracil as first-line chemotherapy for advanced breast cancer. *J Clin Oncol*. **29**, 4498-4504 (2011).

30 Martin, M. et al. Standard versus continuous administration of capecitabine in metastatic breast cancer (GEICAM/2009-05): a randomized, noninferiority phase II trial with a pharmacogenetic analysis. *Oncologist*. **20**, 111-112 (2015).

31 Ciuleanu, E. et al. Capecitabine as salvage treatment in relapsed nasopharyngeal carcinoma: a phase II study. *J BUON*. **13**, 37-42 (2008).

32 Keir, M. E., Butte, M. J., Freeman, G. J. & Sharpe, A. H. PD-1 and its ligands in tolerance and immunity. *Annu Rev Immunol*. **26**, 677-704 (2008).

33 Meng, X., Huang, Z., Teng, F., Xing, L. & Yu, J. Predictive biomarkers in PD-1/PD-L1 checkpoint blockade immunotherapy. *Cancer Treat Rev*. **41**, 868-876 (2015).

34 Hsu, M. C. et al. Increase of programmed death-1-expressing intratumoral CD8 T cells predicts a poor prognosis for nasopharyngeal carcinoma. *Mod Pathol*. **23**, 1393-1403 (2010).

35 Zhang, J. et al. Co-expression of PD-1 and PD-L1 predicts poor outcome in nasopharyngeal carcinoma. *Med Oncol*. **32**, 86 (2015).

36 Lu, J. et al. Detailed analysis of inflammatory cell infiltration and the prognostic impact on nasopharyngeal carcinoma. *Head Neck*. **40**, 1245-1253 (2018).

37 Cao, C. et al. PD-1 and PD-L1 in locoregionally advanced nasopharyngeal carcinoma: A substudy of a randomized phase III trial. *Head Neck* (2018).

38 Brahmer, J. R. et al. Phase I study of single-agent anti-programmed death-1 (MDX-1106) in refractory solid tumors: safety, clinical activity, pharmacodynamics, and immunologic correlates. *J Clin Oncol*. **28**, 3167-3175 (2010).

39 Garon, E. B. et al. Pembrolizumab for the treatment of non-small-cell lung cancer. *N Engl J Med*. **372**, 2018-2028 (2015).

40 Robert, C. et al. Anti-programmed-death-receptor-1 treatment with pembrolizumab in ipilimumab-refractory advanced melanoma: a randomised dose-comparison cohort of a phase 1 trial. *Lancet*. **384**, 1109-1117 (2014).

41 Wolchok, J. D. et al. Nivolumab plus ipilimumab in advanced melanoma. *N Engl J Med*. **369**, 122-133 (2013).

42 J, G. A phase I study of JS001, a humanized IgG4 mAb against programmed death-1 (PD-1) in

patients with advanced solid tumors. *ASCO Abstract* (2017).

43 KY, S. A phase 1 trial of JS001, a monoclonal antibody targeting programmed death-1 (PD-1) in patients with advanced or recurrent malignancies. *ASCO Abstract* (2017).

44 RH, X. Recombinant humanized anti-PD-1 monoclonal antibody (JS001) as salvage treatment for advanced gastric adenocarcinoma: Preliminary results of an open-label, multi-cohort, phase Ib/II clinical study. *ASCO Abstract* (2018).

45 RH, X. Recombinant humanized anti-PD-1 monoclonal antibody (JS001) as salvage treatment for advanced esophageal squamous cell carcinoma: Preliminary results of an open-label, multi-cohort, phase Ib/II clinical study. *ASCO Abstract* (2018).

46 Tang, B. et al. Safety and clinical activity with an anti-PD-1 antibody JS001 in advanced melanoma or urologic cancer patients. *J Hematol Oncol*. **12**, 7 (2019).

47 RH, X. Recombinant humanized anti-PD-1 monoclonal antibody (JS001) in patients with refractory/metastatic nasopharyngeal carcinoma: Preliminary results of an open-label phaseII clinical study. *EMSO Oral* (2018).

48 Jung, S. H., Lee, T., Kim, K. & George, S. L. Admissible two-stage designs for phase II cancer clinical trials. *Stat Med*. **23**, 561-569 (2004).

49 Simon, R. Optimal two-stage designs for phase II clinical trials. *Control Clin Trials*. **10**, 1-10 (1989).
