# Supplementary material for: Toripalimab plus capecitabine in the treatment of patients with residual nasopharyngeal carcinoma: a single-arm phase 2 trial
Source: Nat Commun. 2024 Jan 31;15:949. doi: 10.1038/s41467-024-45276-1 (PMC10831082; doi:10.1038/s41467-024-45276-1)
Supplement: Supplementary file 3 — Reporting Summary [file 41467_2024_45276_MOESM3_ESM.pdf]

nature portfolio

Corresponding author(s): Xing Lv

Last updated by author(s): Jan 5, 2024

Reporting Summary

Nature Portfolio wishes to improve the reproducibility of the work that we publish. This form provides structure for consistency and transparency in reporting. For further information on Nature Portfolio policies, see our Editorial Policies and the Editorial Policy Checklist.

Please do not complete any field with "not applicable" or n/a. Refer to the help text for what text to use if an item is not relevant to your study.
For final submission: please carefully check your responses for accuracy; you will not be able to make changes later.

Statistics

For all statistical analyses, confirm that the following items are present in the figure legend, table legend, main text, or Methods section.

n/a	Confirmed	
☐	☒	The exact sample size (*n*) for each experimental group/condition, given as a discrete number and unit of measurement
☒	☐	A statement on whether measurements were taken from distinct samples or whether the same sample was measured repeatedly
☐	☒	The statistical test(s) used AND whether they are one- or two-sided<br>*Only common tests should be described solely by name; describe more complex techniques in the Methods section.*
☐	☒	A description of all covariates tested
☒	☐	A description of any assumptions or corrections, such as tests of normality and adjustment for multiple comparisons
☐	☒	A full description of the statistical parameters including central tendency (e.g. means) or other basic estimates (e.g. regression coefficient) AND variation (e.g. standard deviation) or associated estimates of uncertainty (e.g. confidence intervals)
☐	☒	For null hypothesis testing, the test statistic (e.g. F, t, r) with confidence intervals, effect sizes, degrees of freedom and P value noted<br>*Give P values as exact values whenever suitable.*
☒	☐	For Bayesian analysis, information on the choice of priors and Markov chain Monte Carlo settings
☒	☐	For hierarchical and complex designs, identification of the appropriate level for tests and full reporting of outcomes
☒	☐	Estimates of effect sizes (e.g. Cohen's *d*, Pearson's *r*), indicating how they were calculated

Our web collection on statistics for biologists contains articles on many of the points above.

Software and code

Policy information about availability of computer code

Data collection	The Patient Electronic Medical Records System (Realone, Neusoft [version 2021.12]).
Data analysis	SPSS (version 26.0), and R (version 4.0.2)

For manuscripts utilizing custom algorithms or software that are central to the research but not yet described in published literature, software must be made available to editors and reviewers. We strongly encourage code deposition in a community repository (e.g. GitHub). See the Nature Portfolio guidelines for submitting code & software for further information.

Data

Policy information about availability of data

All manuscripts must include a data availability statement. This statement should provide the following information, where applicable:

- Accession codes, unique identifiers, or web links for publicly available datasets
- A description of any restrictions on data availability
- For clinical datasets or third party data, please ensure that the statement adheres to our policy

All requests for data will be reviewed by the clinical site Sun Yat-sen University Cancer Centre and the study sponsor, Shanghai Junshi Biosciences Co., to verify if the request is subject to any intellectual property or confidentiality obligations. Data are available to request 12 months after the publication of this article. Requests for access to the De-identified participant data from this study can be submitted via email to lvxing@sysucc.org.cn with detailed proposal for approval. Please allow one month for response to requests. A signed data access agreement with the sponsor is required before accessing the shared data.

Research involving human participants, their data, or biological material

Policy information about studies with human participants or human data. See also policy information about sex, gender (identity/presentation), and sexual orientation and race, ethnicity and racism.

Reporting on sex and gender	In this trial, the sex was determined based on self-reporting. The sex distribution was 15 (65.2%) men versus 8 (34.8%) women. this data was collected in Sun Yat-sen University Cancer Centre and presented in the table of characteristics.
Reporting on race, ethnicity, or other socially relevant groupings	n/a
Population characteristics	The median age of the 23 patients was 52 years (IQR 38-54), and they were mostly men (15 patients [65.2%]). Of the 23 patients, 22 patients (95.7%) received induction chemotherapy plus concurrent chemoradiotherapy. The most common locations of the residual nasopharyngeal carcinoma were nasal skull base (five patients [21.7%]) and cervical lymph nodes (five patients [21.7%]). The detectable and undetectable plasma Epstein–Barr virus DNA were obtained in 2 patients (8.7%) and 21 patients (91.3%), respectively. Ten patients (43.5%) had pathological or cytological diagnoses; and 13 patients (56.5%) had radiological diagnoses.
Recruitment	Investigators in Sun Yat-sen University Cancer Centre screened and enrolled participants into this trial. Participants must meet the all the inclusion criteria and should not meet any one of the exclusion criteria as defined in the protocol. All participants provided written informed consent before study entry.
Ethics oversight	The Institutional Ethics Review Board of Sun Yat-sen University Cancer Centre approved the trial protocol.

Note that full information on the approval of the study protocol must also be provided in the manuscript.

Field-specific reporting

Please select the one below that is the best fit for your research. If you are not sure, read the appropriate sections before making your selection.

☒ Life sciences ☐ Behavioural & social sciences ☐ Ecological, evolutionary & environmental sciences

For a reference copy of the document with all sections, see nature.com/documents/nr-reporting-summary-flat.pdf

Life sciences study design

All studies must disclose on these points even when the disclosure is negative.

Sample size	n=23<br>The sample size was estimated according to Simon's two-stage design with a one-sided α error of 0.025 and a power of 80%.34,35 A previous trial reported that the highest objective response rate of capecitabine monotherapy for recurrent nasopharyngeal carcinoma was 47.8%.28 The objective response rate to the combination regimen (toripalimab plus capecitabine) was initially expected to be 80%. Under these assumptions, the stages were as follows: in stage one, among six evaluable patients, if the responders were three or fewer, the trial would be terminated. Otherwise, an additional 15 patients would be enrolled for stage two. In stage two, if 15 responders or more were observed (including those from stage one), the trial would be considered a success. Assuming a 10% dropout rate, a total of 23 patients were required for this trial.
Data exclusions	Two patients were excluded from our trial (one patient withdrew consent; and one patient received capecitabine monotherapy). The full exclusion criteria are presented in the protocol.
Replication	This is a prospective, single-arm, open-label, phase 2 trial. Our finding can not be reproduced.
Randomization	No randomization. This was a single-arm, phase 2 trial with no comparison arm.
Blinding	No blinding. This was a open-label, phase 2 trial.

Reporting for specific materials, systems and methods

We require information from authors about some types of materials, experimental systems and methods used in many studies. Here, indicate whether each material, system or method listed is relevant to your study. If you are not sure if a list item applies to your research, read the appropriate section before selecting a response.

Materials & experimental systems

n/a	Involved in the study
☒	☐ Antibodies
☒	☐ Eukaryotic cell lines
☒	☐ Palaeontology and archaeology
☒	☐ Animals and other organisms
☐	☒ Clinical data
☒	☐ Dual use research of concern
☒	☐ Plants

Methods

n/a	Involved in the study
☒	☐ ChIP-seq
☒	☐ Flow cytometry
☒	☐ MRI-based neuroimaging

Clinical data

Policy information about clinical studies

All manuscripts should comply with the ICMJE guidelines for publication of clinical research and a completed CONSORT checklist must be included with all submissions.

Clinical trial registration: The trial is registered with the Chinese Clinical Trial Registry, number ChiCTR1900023710

Study protocol: Attached in our manuscript

Data collection: This trial was performed at SunYat-sen University Cancer Centre in Guangzhou, China. Patients were recruited between June 1, 2020, and May 31, 2021.

Outcomes: The primary endpoint of this trial was the objective response rate. The following secondary endpoints were also analysed: complete response rate, disease control rate, duration of response, progression-free survival, safety profile and treatment compliance.
The objective response rate, complete response rate and disease control rate were assessed by an independent review team according to RECIST (version 1.1); the accompanying 95% confidence intervals (95% CIs) were calculated based on the Clopper-Pearson method. The median duration of response and median progression-free survival were estimated using the Kaplan–Meier method, and the corresponding 95% CIs were estimated using the Brookmeyer-Crowley method.
The efficacy was assessed by an independent review team according to NCI CTC-AE (version 5.0).

Plants

Seed stocks: *Report on the source of all seed stocks or other plant material used. If applicable, state the seed stock centre and catalogue number. If plant specimens were collected from the field, describe the collection location, date and sampling procedures.*

Novel plant genotypes: *Describe the methods by which all novel plant genotypes were produced. This includes those generated by transgenic approaches, gene editing, chemical/radiation-based mutagenesis and hybridization. For transgenic lines, describe the transformation method, the number of independent lines analyzed and the generation upon which experiments were performed. For gene-edited lines, describe the editor used, the endogenous sequence targeted for editing, the targeting guide RNA sequence (if applicable) and how the editor was applied.*

Authentication: *Describe any authentication procedures for each seed stock used or novel genotype generated. Describe any experiments used to assess the effect of a mutation and, where applicable, how potential secondary effects (e.g. second site T-DNA insertions, mosiacism, off-target gene editing) were examined.*

This checklist template is licensed under a Creative Commons Attribution 4.0 International License, which permits use, sharing, adaptation, distribution and reproduction in any medium or format, as long as you give appropriate credit to the original author(s) and the source, provide a link to the Creative Commons license, and indicate if changes were made. The images or other third party material in this article are included in the article's Creative Commons license, unless indicated otherwise in a credit line to the material. If material is not included in the article's Creative Commons license and your intended use is not permitted by statutory regulation or exceeds the permitted use, you will need to obtain permission directly from the copyright holder. To view a copy of this license, visit http://creativecommons.org/licenses/by/4.0/
